# RNA Sequencing Reveals the Alteration of the Expression of Novel Genes in Ethanol-Treated Embryoid Bodies

**DOI:** 10.1371/journal.pone.0149976

**Published:** 2016-03-01

**Authors:** Chanchal Mandal, Sun Hwa Kim, Jin Choul Chai, Seon Mi Oh, Young Seek Lee, Kyoung Hwa Jung, Young Gyu Chai

**Affiliations:** 1 Department of Molecular and Life Science, Hanyang University, Ansan, Republic of Korea; 2 Institute of Natural Science and Technology, Hanyang University, Ansan, Republic of Korea; 3 Department of Bionanotechnology, Hanyang University, Seoul, Republic of Korea; National Renewable Energy Lab, UNITED STATES

## Abstract

Fetal alcohol spectrum disorder is a collective term representing fetal abnormalities associated with maternal alcohol consumption. Prenatal alcohol exposure and related anomalies are well characterized, but the molecular mechanism behind this phenomenon is not well characterized. In this present study, our aim is to profile important genes that regulate cellular development during fetal development. Human embryonic carcinoma cells (NCCIT) are cultured to form embryoid bodies and then treated in the presence and absence of ethanol (50 mM). We employed RNA sequencing to profile differentially expressed genes in the ethanol-treated embryoid bodies from NCCIT vs. EB, NCCIT vs. EB+EtOH and EB vs. EB+EtOH data sets. A total of 632, 205 and 517 differentially expressed genes were identified from NCCIT vs. EB, NCCIT vs. EB+EtOH and EB vs. EB+EtOH, respectively. Functional annotation using bioinformatics tools reveal significant enrichment of differential cellular development and developmental disorders. Furthermore, a group of 42, 15 and 35 transcription factor-encoding genes are screened from all of the differentially expressed genes obtained from NCCIT vs. EB, NCCIT vs. EB+EtOH and EB vs. EB+EtOH, respectively. We validated relative gene expression levels of several transcription factors from these lists by quantitative real-time PCR. We hope that our study substantially contributes to the understanding of the molecular mechanism underlying the pathology of alcohol-mediated anomalies and ease further research.

## Introduction

Prenatal exposure to alcohol has profound effects on many aspects of fetal development. Although alterations in somatic growth and specific minor malformations of facial structure are most characteristic, the effects of alcohol on brain development are most significant in that they lead to substantial problems with neurobehavioral development. Since the initial recognition of the fetal alcohol syndrome (FAS), a number of important observations have been made from studies involving both humans and animals. Of particular importance, a number of maternal risk factors have been identified, which may be of relevance in the development of strategies for the prevention of the FAS and intervention for those who have been affected.

In recent decades, tremendous progress has been achieved in the research area related to alcoholic toxicity during fetal development. Alcohol can cause dramatic and irreversible effects on the fetus, such as developmental delay, head and facial irregularities, seizures, hyperactivity, attention deficits, cognitive deficits, learning and memory impairments, poor psychosocial functioning, facial irregularities, and motor coordination deficits [1]. However, the exact developmental phases in which alcohol has these specific effects on the fetus are not entirely known. Several findings related to molecular mechanism have been published recently, including studies implicating retinoic acid signaling [2,3,4], glucocorticoid signaling [4,5] stress response genes [6,7], mitogen-activated protein kinase (MAPK) cascade [8], neurotransmitters [9,10], phosphoinositide 3-kinase [11], calcium signaling [12], Wnt signaling [13,14], and the Notch and JAK/STAT signaling pathways [11,15].

Epigenetic modifications, including DNA methylation in particular, regulate key developmental processes, including germ cell imprinting and stem cell maintenance/differentiation, and play a crucial role in the early periods of embryogenesis [16,17,18]. DNA methylation is also a fundamental aspect of programmed fetal development, determination of cell fate, pattern formation, terminal differentiation and maintenance of cellular memory required for developmental stability [17,19]. Moreover, aberrant epigenetic changes in response to environmental stimuli have been shown to contribute to developmental disorders [20]. Recently, several hypotheses involving alcohol (ethanol)-induced changes in genetic and epigenetic regulation of cells as possible molecular mechanisms of fetal alcohol spectrum disorders (FASDs) have been advanced [21,22,23,24,25,26]. However, the precise mechanisms by which ethanol alters the transcriptional landscape are still largely unknown. In addition, ethanol influences the molecular, cellular, and physiological regulation of adult stem cells in a dose-dependent manner, which likely contributes to the deleterious consequences of excessive alcohol consumption in adults [27,28,29,30].

Embryonic carcinoma (EC) cells exhibit pluripotent gene expression profiles similar to embryonic stem cells and both of these cell types exhibit unlimited self-renewal capacity and can give rise to derivatives of all three embryonic germ layers as demonstrated by EBs in cell culture and in the development of tumors after injection into adult mice [31,32]. EC cells are derived from malignant teratocarcinoma and can proliferate independently of growth factors and cytokines. In vitro, EBs can differentiate spontaneously from ES, EG and EC cells as aggregates. EBs are part of a well-established model to investigate cellular differentiation and gene expression patterns during ES, EC and EG cell differentiation in vitro [33]. In the present study, we have set up an in vitro model for ethanol exposure on NCCIT cell-derived embryoid bodies (EBs), which mimic early fetal development. Our goal was to profile novel genes that were altered by ethanol to aid future research regarding alcohol-related fetal abnormalities. We compare the gene expression profiles in the presence and absence of ethanol by RNA sequencing analysis. We also carried out extensive bioinformatics analysis on the gene expression data and selected a group of genes that encode transcription factors (TFs) during embryonic development.

## Materials and Methods

### Cell Culture and Ethanol Treatment

**Fig 1:**
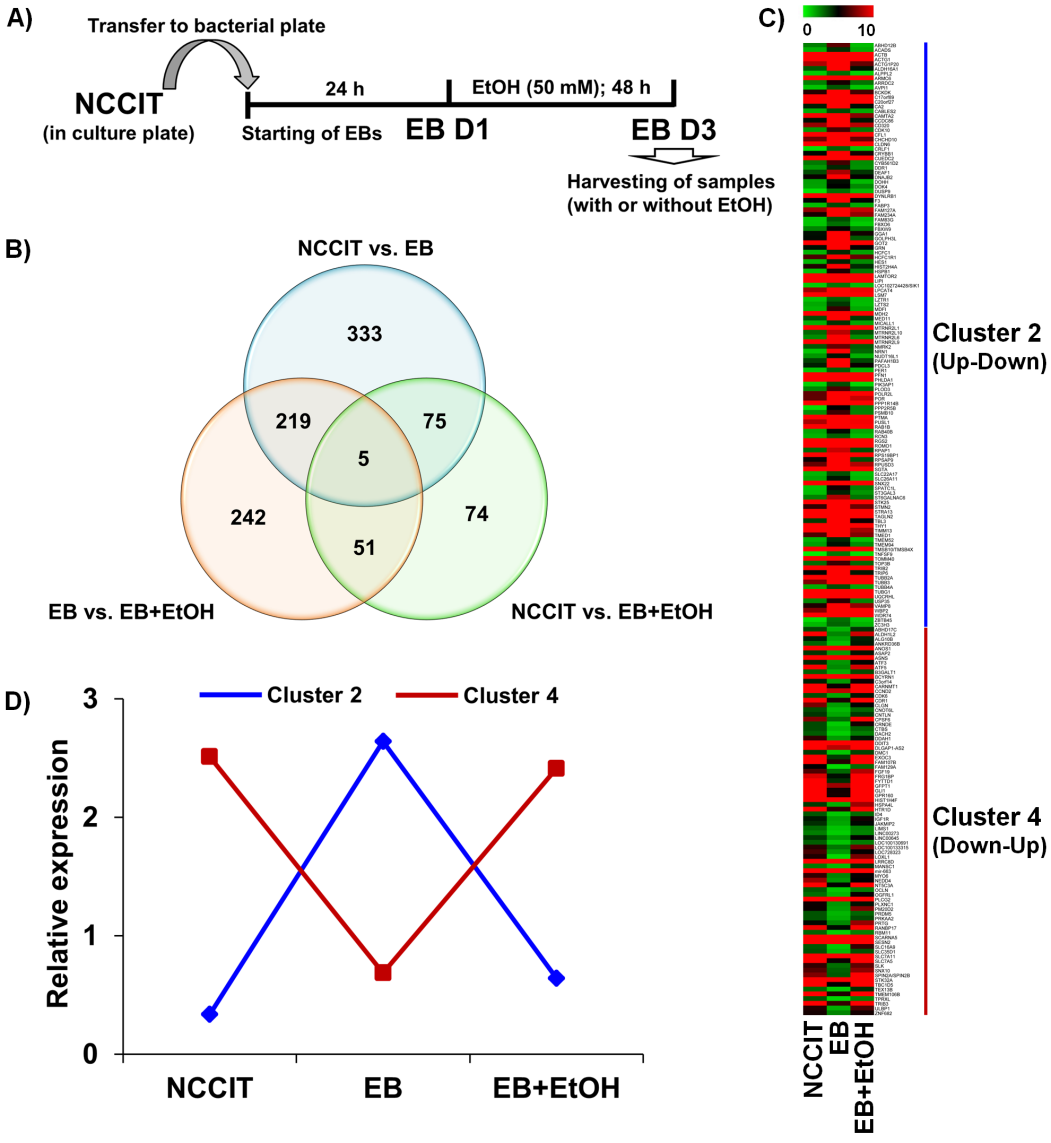
Functional annotation of differential gene expression. A) Graphical experimental scheme for differentiation/treatment protocol. NCCIT cells were stabilized and subcultured to form EBs. After stabilization, the EBs were treated with or without EtOH for 48 h. The samples were then collected for further analyses. B) Venn diagram representing the overall distribution of all differentially expressed genes. A total of 632, 205 and 517 differentially expressed genes were identified from NCCIT vs. EB, NCCIT vs. EB+EtOH and EB vs. EB+EtOH, respectively. C) Heat map representing all DEGs that found common between NCCIT vs. EB and EB vs. EB+EtOH datasets. Genes with specific expression patterns were clustered into 4 groups–cluster 1, 2, 3 and 4 represent up-up, up-down, down-down and down-up, respectively. Cluster 2 and 4 were listed here. D) Line graph representing relative expression pattern of those gene clusters defined from the transcriptomic profiling. Genes with specific expression patterns were clustered according to their relative expression values.

We used human embryonic carcinoma (NCCIT), collected from the American Type Culture Collection (CRL-2073) cells for our study. The cells were cultured in RPMI-1640 media supplemented with 10% fetal bovine serum (FBS), 100 IU ml–1 penicillin and 10 μg ml–1 streptomycin at 37°C in a CO_2_ incubator. For EB formation, 1 × 10^6^ NCCIT cells were plated in the 90-mm bacterial culture dishes (non-adherent culture conditions) in Opti-MEM growth medium for 24 h. The morphology of EBs was examined by phase contrast microscope and immunocytochemistry analysis (S1 Fig). After 24 h, the EBs were exposed to ethanol for another 48 h, whereas control cells remained untreated. We changed culture media every day to ensure a constant ethanol concentration during the course of the study. To prevent ethanol evaporation from the culture dishes, ethanol-treated cells were cultured in a separate CO_2_ incubator that was saturated with 50 mM ethanol, as previously described by our group [34]. A graphical experimental scheme of our protocol is presented in Fig 1A.

### Isolating Total RNA and cDNA Synthesis

Total RNA samples were extracted by homogenization in RNAiso Plus (Takara BIO, Shiga, Japan) according to the manufacturer instructions. Briefly, after mixing of chloroform (200 ml) it was gently inverted for 5 minutes and then centrifuged for 15 min at 14,000 x g at 4°C. The upper solution was collected and 600 μl of isopropanol was added to it. After 1 h incubation the lysis mixture was centrifuged at 14,000 x g for 15 min at 4°C, and the isopropanol was decanted. Ice-cold 70% ethanol was added to the RNA pellet for gentle washing. After centrifuging for 10 min, the ethanol was discarded. The RNA pellets were dried at room temperature for 5 min and then added 20 μl RNase-free water in it. The quality and quantity of extracted total RNA was measured by an Agilent 2100 Bioanalyzer (Agilent Technologies, Waldbronn, Germany) and a spectrophotometer (NanoDrop Technologies, Wilmington, DE, USA), respectively. Reverse transcription of the extracted RNA was conducted according to Halder et al., 2014[2]. In brief, the first-strand cDNA was synthesized with SuperScript II Reverse Transcriptase (Invitrogen, Carlsbad, CA, USA).

### RNA Sequencing (RNA-seq)

Ribosomal RNAs (rRNAs) from total RNA (5 μg) were removed using a RiboMinusTM Transcriptome Isolation Kit (Invitrogen). rRNA-depleted total RNAs (100 ng) were used to construct paired-end transcriptome libraries using the NEBNext® UltraTM Directional RNA Library Prep Kit for Illumina® (New England Biolabs, Ipswich, MA, USA). Briefly, first-strand cDNAs were synthesized from rRNA-depleted RNA samples, followed by second-strand synthesis with DNA polymerase I and RNase H. The double-stranded cDNAs were then end-repaired and ligated to adaptors. Ligated libraries were then separated on a 2% agarose gel (Duchefa, Haarlem, The Netherlands), and fragments with sizes between 300–400 bp were purified using the MinElute Gel Extraction Kit (Qiagen, Hilden, Germany). Fragments were amplified for further enrichment and purified with ethanol precipitation. The cDNA fragments (101 bp) were sequenced using the Illumina HiSeq2500 (101 cycles PE lane) (National Instrumentation Center for Environmental Management in Seoul National University). Two biological replicates were prepared from each condition. A tabular description of read count is presented in the S1 Table. Gene expression data have been submitted to the NCBI Sequence Read Archive (SRA) repository (http://www.ncbi.nlm.nih.gov/sra/) under accession numbers SRX904625, SRX904869 and SRX1175001.

### Analysis of the Sequencing Data

Raw sequence files underwent a quality control analysis using FastQC [35]. To avoid low-quality data, we clipped the adapters and trimmed the reads using FASTX-Toolkit [36]. Paired-end reads were alignments to the Homo sapience genome (Homo sapiens UCSC hg19) using Top hat [37] using default parameter. For the analysis of differential expressed analysis was performed with Cufflinks [38]. Default settings were used in aforementioned methods. Differentially expressed genes showing more than 1.0-fold change in their log_2_ fold-change (*P*-value < 0.05) were selected for functional annotation.

### Functional Annotation, Heat Map Construction and Enrichment Analysis

Functional annotation of significant genes identified by the RNA-seq analysis was searched using the web accessible program Database for Annotation, Visualization and Integrated Discovery (DAVID) (http://david.abcc.ncifcrf.gov). DAVID calculates a modified Fisher's Exact *P*-value to demonstrate gene ontology (GO), where *P*-values less than 0.05 are considered to be strongly enriched in the annotation category. We constructed heat maps to view the relative expression patterns of our array data using TIGR Multiexperiment Viewer (MeV). MeV is a Java-based microarray data analysis tool (desktop application) that allows advanced analysis of gene expression data through an intuitive graphical user interface. We uploaded our array data in text file format and chose two color arrays to create heat maps. Integrated disease enrichment analysis was performed using ingenuity pathway analysis (IPA). The DEGs were mapped in the analysis tool and observed for significant disease pathway enrichment. This analysis helped us understand causal connections between diseases and genes.

### Quantitative Reverse Transcription-Polymerase Chain Reaction

All assays were run on a ABI 7500 Real-Time PCR System (Applied Biosystems, Inc., Foster City, CA, USA) using the SYBR Premix Ex Taq^TM^ II (Otsu-Shi, Shiga, Japan). The reaction volume was 20 μL and the PCR conditions were as follows: 30 sec. at 95°C, 40 cycles of 5 sec. at 95°C and 34 sec. at 60°C, followed by a melting curve analysis step. If all amplicon showed a single Tm, the PCR reactions were considered specific. Every sample was measured in triplicate, and relative quantification was effected by means of the comparative CT (ΔΔCT) method. *GAPDH* was used as a housekeeping gene to normalize the expression data. The primers used for gene validation are listed in S2 Table.

### Transcription Factor Binding Motif Enrichment Analysis

NCBI reference sequence mRNA accession numbers were subjected to transcription factor binding motif analysis using the web-based software Pscan (http://159.149.109.9/pscan/) [39]. The JASPAR [40] database of transcription binding factor sequences was analyzed using enriched groups of −950 base pair (bp) sequences to +50 bp of the 5’ upstream promoters. The range of −950 to +50 was selected from the range options in Pscan to obtain the best cover for a −1000 to +50 bp range.

### Statistical Analysis

In this study, we ran three technical replicates to study the relative gene expressions for control and treated samples. For qRT-PCR analysis, results are presented as the mean ± standard error of the mean (SEM) (n = 3). For the statistical analyses, Student’s t-test was performed using the Microsoft Office Excel, 2010 program at the 0.05 probability level.

## Results

### Gene Expression Profile Analysis of Ethanol Exposure During Early Development

We performed RNA-seq analysis for NCCIT, EB and EB with EtOH and compared each dataset with another in the manner of NCCIT vs. EB, NCCIT vs. EB+EtOH and EB vs. EB+EtOH. After normalization of gene expression profiling data, we first examined the number of all altered genes. Using the threshold of more than 1.0-fold change in their log_2_ fold-change (*P*-value < 0.05), we identified 632, 205 and 517 differentially expressed genes (DEGs) in NCCIT vs. EB, NCCIT vs. EB+EtOH and EB vs. EB+EtOH datasets, respectively. NCCIT vs. EB+EtOH and EB vs. EB+EtOH datasets represent DEGs that were altered in response to acute alcohol intoxication. In the NCCIT vs. EB+EtOH dataset 122 genes were up-regulated and 83 were down-regulated whereas, 213 and 304 genes were up- and down-regulated in the EB vs. EB+EtOH dataset, respectively. A total of 75, 219 and 51 genes are common between NCCIT vs. EB and NCCIT vs. EB+EtOH, NCCIT vs. EB and EB vs. EB+EtOH, and NCCIT vs. EB+EtOH and EB vs. EB+EtOH, respectively. The number of altered genes showed a clear view of the deteriorative effect of ethanol during early development. To observe a clear comparison between these 3 datasets we have drawn a Venn diagram where intersectional comparisons were represented in a 2 by 2 comparison manner (Fig 1B). We have constructed a heat map that represented all common DEGs between NCCIT vs. EB and EB vs. EB+EtOH. We have divided all DEGs into 4 clusters where Cluster 1, 2, 3 and 4 represent up-up, up-down, down-down and down-up relationships between the compared datasets, respectively. Unfortunately, we did not get any genes under cluster 1 and 3 where 124 and 82 genes were listed under cluster 2 and 4, respectively (Fig 1C). We also drew line graph to generalize the expression patterns of the DEGs among these datasets (Fig 1D). The numbers of altered genes represent the effect of alcohol intoxication during early fetal development which may have a serious consequences regarding proper embryonic development.

### Pathway Analysis of Differentially Expressed Genes

To obtain a global view of the biological processes represented in these DEGs, we carried out GO term enrichment analysis using a false discovery rate (FDR) cutoff of 0.05. We have short-listed the total enriched GO terms and showed in the S2 Fig. To explore the ethanol responsive categories we considered the comparison between EB vs. EB+EtOH dataset. GO term enrichment analysis revealed preferential increases in the expression of genes in response to ethanol exposure involved in diverse cellular activities including “positive regulation of cell proliferation”, “carbohydrate biosynthetic process”, “embryonic organ development”, “regulation of cell development”, “negative regulation of cell differentiation” and others (S2C Fig). In addition, there was a dramatic decrease in the levels of transcripts in the GO categories of “intracellular signaling cascade”, “regulation of cell proliferation”, “response to organic substance”, “positive regulation of molecular function”, “negative regulation of signal transduction”, and others between the control and ethanol-treated EBs (S2C Fig).We have also provided the enriched terms for another two sets of comparison groups in S2A Fig and S2B Fig.

Ingenuity pathway analysis (IPA) revealed 71 different enriched canonical pathways (*P*-value < 0.05, at least 5 DEGs listed) from the EB vs. EB+EtOH dataset. Important pathways are ILK signaling, axonal guidance signaling, RhoA signaling, mTOR signaling etc. The top 15 pathways that are significantly enriched to the differentially enriched genes are listed in Table 1. We also listed enriched canonical pathways for other two datasets. In brief, DEGs were significantly involved in a total of 119 and 17 pathways in NCCIT vs. EB and NCCIT vs. EB+EtOH, respectively. Top 15 enriched pathways from each dataset were listed in S3 Table and S4 Table, respectively. The enrichment of different pathways provide a clear idea that ethanol has the ability to alter cell state or to mislead directed paths during early development.

**Table 1:** Top 15 enriched canonical pathways of all differentially expressed genes in the EB vs. EB+EtOH datasets.

Ingenuity Canonical Pathways	-log (*p*-value)	Molecules
Remodeling of epithelial adherens junctions	4.04E00	*TUBB3*, *ACTB*, *ACTN3*, *TUBB2A*, *TUBG1*, *TUBB4A*, *ACTG1*, *MAPRE3*
Sertoli cell-sertoli cell junction signaling	3.37E00	*TUBB3*, *CLDN19*, *ACTB*, *ACTN3*, *TUBB2A*, *TUBG1*, *TUBB4A*, *JUP*, *CLDN6*, *ELK1*, *ACTG1*, *OCLN*
Germ cell-sertoli cell junction signaling	3.21E00	*TUBB3*, *RHOG*, *CFL1*, *RHOB*, *ACTB*, *ACTN3*, *TUBB2A*, *TUBG1*, *TUBB4A*, *JUP*, *ACTG1*
ILK signaling	2.68E00	*FN1*, *RHOG*, *CFL1*, *RHOB*, *LIMS1*, *ACTB*, *ACTN3*, *PPP2R5B*, *TMSB10*, *PPP1R14B*, *ACTG1*
Glioblastoma multiforme signaling	2.4E00	*FZD10*, *PLCB4*, *RHOG*, *RHOB*, *PDGFA*, *PLCG2*, *IGF1R*, *CDK6*, *FZD6*
Epithelial adherens junction signaling	2.4E00	*TUBB3*, *ACTB*, *ACTN3*, *TUBB2A*, *TUBG1*, *ACVR1*, *TUBB4A*, *JUP*, *ACTG1*
Phagosome maturation	2.39E00	*ATP6V0C*, *TUBB3*, *VPS28*, *TUBB2A*, *TUBG1*, *TUBB4A*, *DYNLRB1*, *PRDX2*
Semaphorin signaling in neurons	2.29E00	*CRMP1*, *RHOG*, *CDK5*, *CFL1*, *RHOB*
Gap junction signaling	2.23E00	*PLCB4*, *TUBB3*, *GUCY2C*, *PLCG2*, *ACTB*, *TUBB2A*, *TUBG1*, *TUBB4A*, *ACTG1*
Axonal guidance signaling	1.94E00	*FZD10*, *TUBB3*, *PLXNC1*, *PFN1*, *CFL1*, *PDGFA*, *TUBG1*, *TUBB2A*, *PTCH1*, *PLCB4*, *SEMA6D*, *CDK5*, *NGFR*, *PLCG2*, *FZD6*, *TUBB4A*, *GLI1*
14-3-3-mediated signaling	1.91E00	*PLCB4*, *TUBB3*, *PLCG2*, *TUBB2A*, *TUBG1*, *TUBB4A*, *ELK1*
D-myo-inositol-5-phosphate metabolism	1.9E00	*PLCB4*, *PPP1R1A*, *PLCG2*, *PPP2R5B*, *NUDT14*, *PPP1R14B*, *PXYLP1*, *DUSP14*
RhoA signaling	1.82E00	*NEDD4*, *PFN1*, *CFL1*, *ACTB*, *IGF1R*, *ARHGEF1*, *ACTG1*
Actin cytoskeleton signaling	1.75E00	*FN1*, *PFN1*, *CFL1*, *PDGFA*, *ACTB*, *ACTN3*, *ARHGEF1*, *TMSB10*, *ACTG1*, *FGF19*
PI3K signaling in B lymphocytes	1.72E00	*PLCB4*, *ATF3*, *NFKBIA*, *ATF5*, *PLCG2*, *PIK3AP1*, *ELK1*

### Neuronal Development is Activated by Ethanol During Early Development

**Fig 2:**
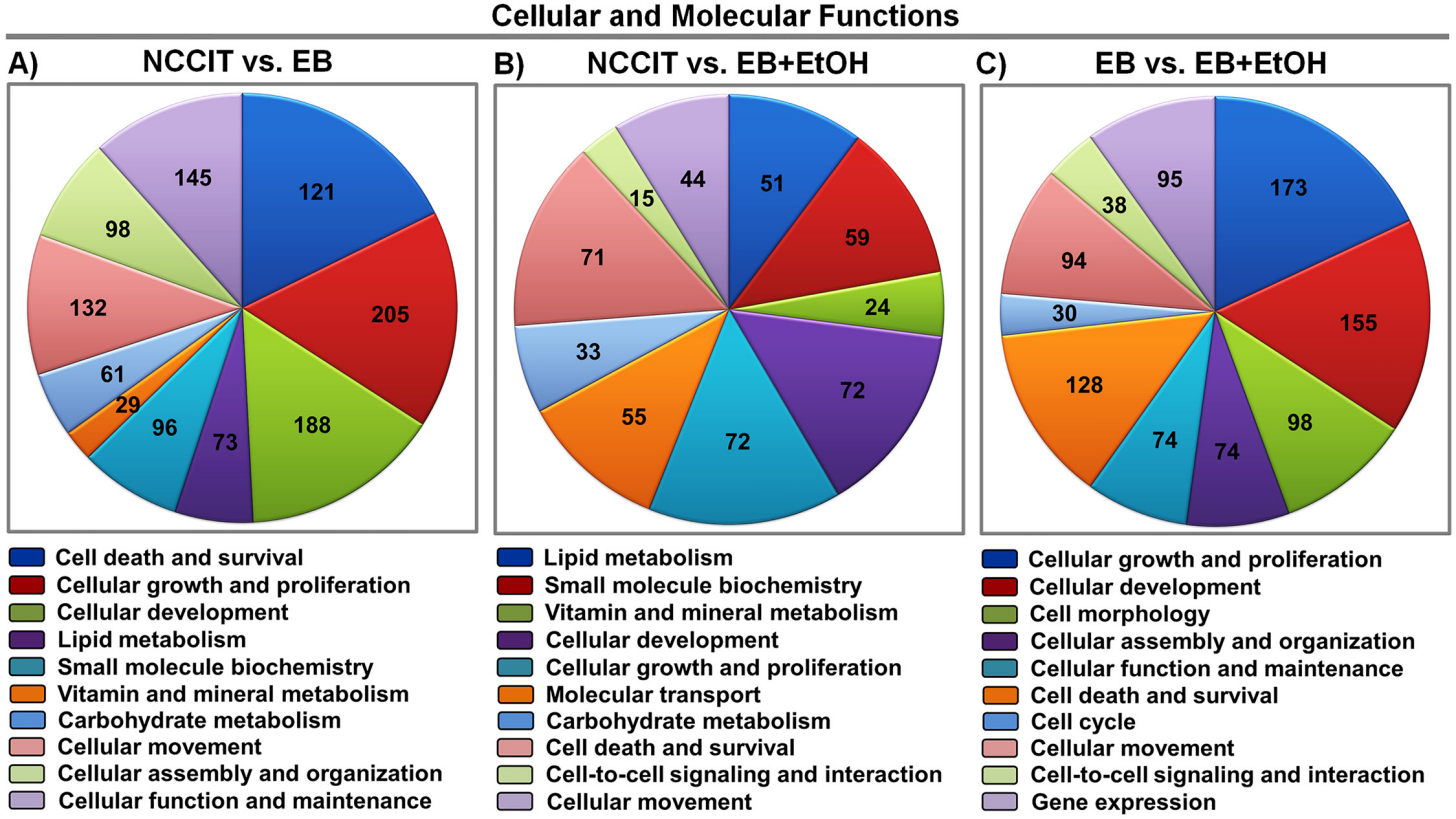
Functional enrichment analysis of differentially expressed genes. A). B) and C) represent Pie charts of cellular and molecular functions of the DEGs (top 10 categories; *p*-values < 0.05) between NCCIT vs. EB, NCCIT vs. EB+EtOH and EB vs. EB+EtOH, respectively. Numbers in the charts represent the relative genes enriched.

From the GO analysis, we gained an idea of functional categories of the DEGs. Again, to obtain more molecular information, we examined our gene list for cellular and molecular functional analysis using IPA. Under the threshold of <0.05, a total of 21 categories in EB vs. EB+EtOH dataset are listed. The second top most enriched category is “cellular development” in which 155 genes are altered by ethanol exposure (Fig 2C). These 155 genes are involved in the development of neurons, proliferation of carcinoma cell lines, differentiation of cells, neuritogenesis, development of central nervous system cells and also other important developmental functions (data not shown). Thus, any alteration of these developmental processes will raise major disorders. We also analyzed enriched categories for NCCIT vs. EB and NCCIT vs. EB+EtOH. In brief, a total of 24 and 25 categories are listed, respectively. The top 10 functions of each comparison group are presented in Fig 2A and 2B.

**Fig 3:**
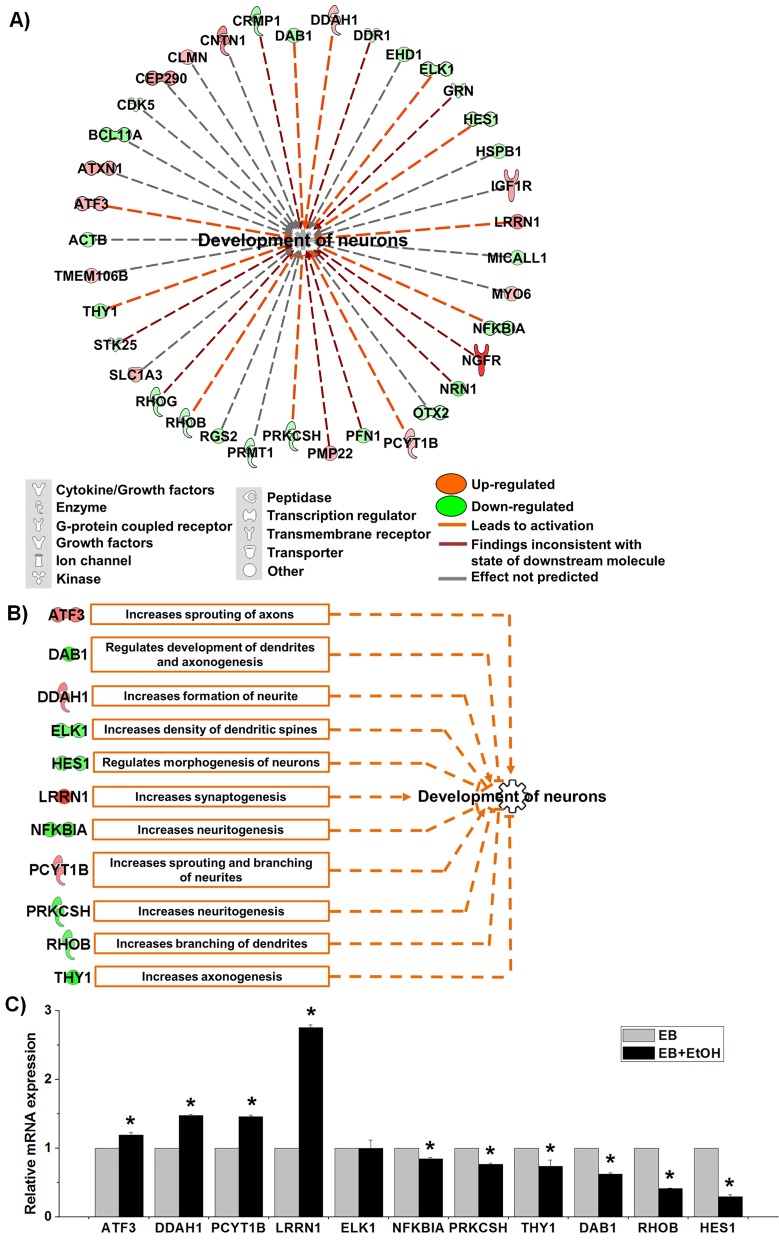
Activation of the neuronal development process by ethanol. A) Network for the category “development of neuron” that was adopted from IPA. The relationship is mentioned at the bottom. B) A functional network analysis of the 11 DEGs that were predicted to activate neuronal development. Boxes represent proposed roles in neuronal development in aspect of expression patterns found in EB vs. EB+EtOH data set. C) qRT-PCR analysis for the relative mRNA expression of the 11 genes responsible for the activation of neuronal development. The expression value was normalized to the GAPDH expression level. Values are represented as average mRNA expression ± SEM bars, n = 3 replicates. Asterisks indicate statistically significant changes based on adjusted *p*-values < 0.05.

From the list shown in Fig 2C we have noticed that a large amount of differentially expressed genes (37) were enriched to “development of neurons”. The findings were interesting and we drew a network for development of neurons and found that 11 genes were involved to activate neuronal development (marked as orange lines in the network, Fig 3A). It was reported that knocking down of rat *Pcyt1b* gene decreased sprouting and branching of neurites in PC-12 cells [41]. Additionally, induced expression of mouse *Lrrn1*, *Ddah1* and *Atf3* increased synaptogenesis [42], increased formation of neurite [43] and increased sprouting of axons [44], respectively. Zine et al. [45] have reported that mouse *Hes1* is involved in differentiation morphogenesis of neurons and decreasing of its expression results increased hair cells formation [46]. Knocking down of mouse *Rhob* gene increased branching of dendrites and increased length of dendritic spines in pyramidal cells [47]. Additionally, knocking down of rat *Elk1* mRNA by shRNA increases density of dendritic spines [48]. DAB1 protein is an important candidate during neural development. It was reported that human DAB1 protein affects formation to neurite in cultured chicken retinal cells [49]. Additionally, mouse *Dab1* is involved in development of dendrites [50] and it decreased axonogenesis of axons [51],same as rat *Thy1* [52]. Furthermore, Yaguchi et al.[53] reported that suppression of mouse *Prkcsh* mRNA by shRNA increased neuritogenesis of N1E-115 cells in cell culture. NF-kappa B signaling promotes both cell survival and neurite process formation in nerve growth factor-stimulated PC12 cells [54]. We have prepared a functional network for these 11 genes only where their proposed roles in neuronal development were plotted (Fig 3B).We validated all of the 11 genes by qRT-PCR. We observed that all of selected genes were significantly altered to activate neuronal development as IPA network claimed except *ELK1*(Fig 3C). Thus, we assumed that there would be an assistance effect exerted by ethanol itself during the development process.

### A Set of Transcription Factor-encoding Genes are Altered by Ethanol

**Fig 4:**
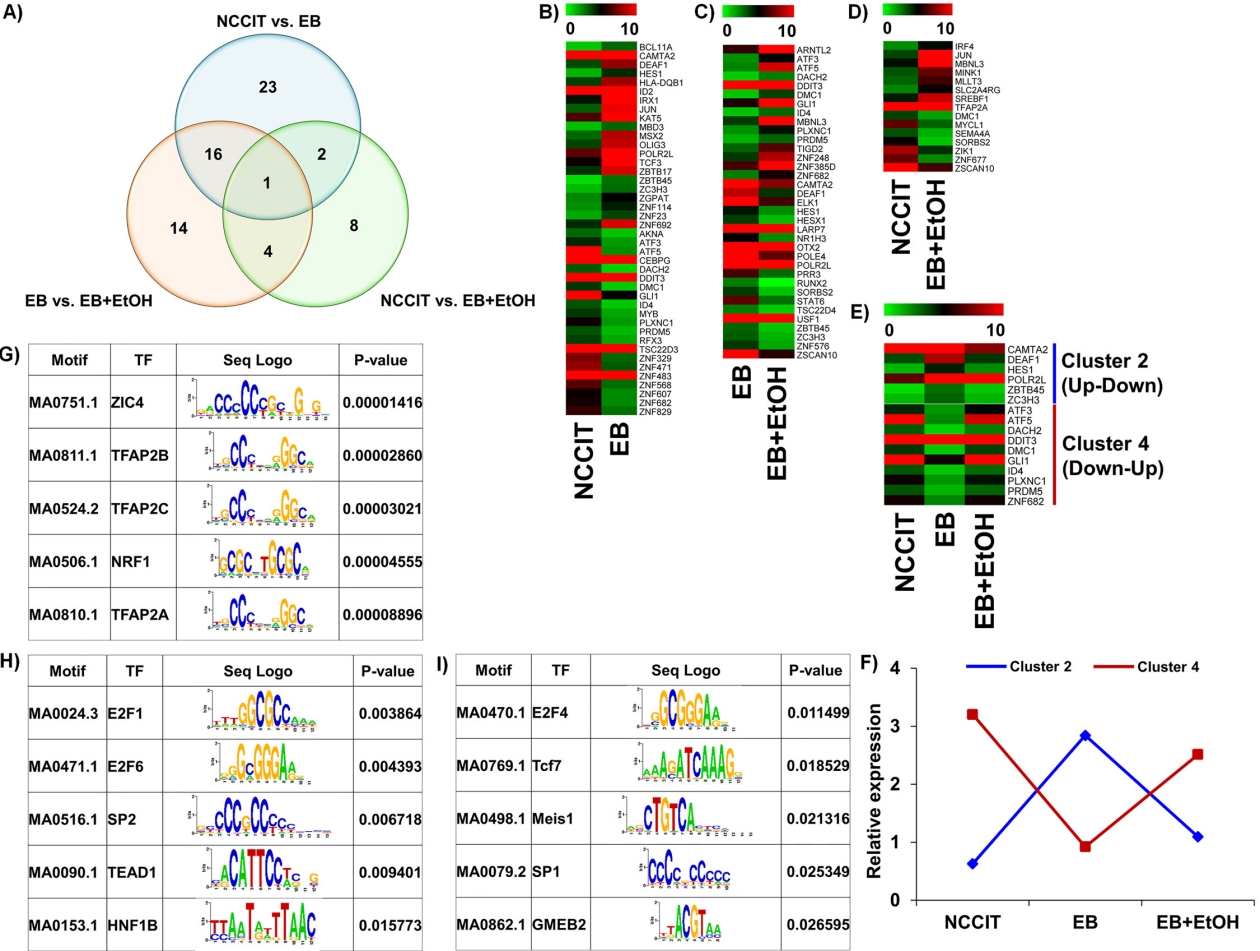
Distribution of all TF-encoding genes that are altered by ethanol. A) Venn diagram representing the overall distribution of all differentially expressed TF-encoding genes. A total of 42, 15 and 35 differentially expressed genes were identified from NCCIT vs. EB, NCCIT vs. EB+EtOH and EB vs. EB+EtOH, respectively. A total of 2,16 and 4 genes were common between NCCIT vs. EB and NCCIT vs. EB+EtOH, NCCIT vs. EB+EtOH and EB vs. EB+EtOH, and NCCIT vs. EB and EB vs. EB+EtOH, respectively. B), C) and D) represent heat maps of differential TF-encoding gene expressions between NCCIT vs. EB, EB vs. EB+EtOH and NCCIT vs. EB+EtOH, respectively. Gene expression level of each gene in the heat map is scaled and represented as relative expression value. E) Represents heat maps for TF-encoding genes found common between NCCIT vs. EB and EB vs. EB+EtOH. Genes with specific expression patterns were clustered into 4 groups–cluster 1, 2, 3 and 4 represent up-up, up-down, down-down and down-up, respectively. Only up-down and down-up relationships were found enriched. F) Line graph representing relative expression pattern of those TF-encoding gene clusters defined from the transcriptomic profiling. Genes with specific expression patterns were clustered according to their relative expression values. G), H) and I) represent transcription motif analysis of selected 42, 15 and 35 TF-encoded genes, respectively. Significantly enriched top 5 motifs are presented here (*p*-value < 0.05). The sequence logos are illustrated in the third column of each table.

At this stage we were prompted to profile ethanol-responsive TFs available in our datasets. To verify the group of ethanol-targeted TFs, we compared our DEGs with a list of human TFs provided by Vaquerizas *et al*., 2009 [55]. We searched for TFs in our all datasets and identified a huge number of TFs that are ethanol responsive. We have listed a total of 42 TFs in NCCIT vs. EB, a total of 15 TFs in NCCIT vs. EB+EtOH and a total of 35 TFs in EB vs. EB+EtOH dataset. The number of altered TF-encoding genes showed a clear view of the deteriorative effect of ethanol during early development. All identified TFs are listed in Table 2. To observe a clear comparison between these 3 datasets we have drawn a Venn diagram where intersectional comparisons were represented in a 2 by 2 comparison manner (Fig 4A). Based on the 1.0 log_2_ fold-change criteria for mining the biological data, heat maps were constructed where the expression changes were detected very easily (Fig 4B, 4C and 4D). We also drew heat maps for common TF-encoding genes between NCCIT vs. EB and EB vs. EB+EtOH datasets (Fig 4E).We have divided all differentially expressed TFs into 4 clusters where Cluster 1, 2, 3 and 4 represent up-up, up-down, down-down and down-up relationships between the compared datasets, respectively. Unfortunately, we did not get any genes under cluster 1 and 3 where 6 and 10 genes were listed under cluster 2 and 4, respectively (Fig 4E). We also drew line graph to generalize the expression patterns of the common differentially expressed TFs among these datasets (Fig 4F).The relative expression differences were clearly noticeable in those figures.

**Table 2:** List of TFs that were differentially expressed during early development.

GeneBank accession no.	Gene symbol	Gene description	Log_2_(Fold change)
NCCIT vs. EB			
NM_018014	*BCL11A*	B-cell CLL/lymphoma 11A (zinc finger protein)	1.46762
NM_001171166	*CAMTA2*	Calmodulin binding transcription activator 2	1.04657
NM_021008	*DEAF1*	DEAF1 transcription factor	1.17328
NM_005524	*HES1*	Hes family bHLH transcription factor 1	1.52674
NM_001243961	*HLA-DQB1*	Major histocompatibility complex, class II, DQ beta 1	1.14242
NM_002166	*ID2*	Inhibitor of DNA binding 2, dominant negative helix-loop-helix protein	1.53728
NM_024337	*IRX1*	Iroquois homeobox 1	1.36066
NM_002228	*JUN*	JUN proto-oncogene	1.61828
NM_001206833	*KAT5*	K(lysine) acetyltransferase 5	1.05922
NM_001281453	*MBD3*	Methyl-CpG binding domain protein 3	1.21166
NM_002449	*MSX2*	Msh homeobox 2	1.55794
NM_175747	*OLIG3*	Oligodendrocyte transcription factor 3	1.28199
NM_021128	*POLR2L*	Polymerase (RNA) II (DNA directed) polypeptide L, 7.6kDa	1.69433
NM_001136139	*TCF3*	Transcription factor 3	1.03986
NM_001242884	*ZBTB17*	Zinc finger and BTB domain containing 17	1.21244
NM_032792	*ZBTB45*	Zinc finger and BTB domain containing 45	2.10383
NM_015117	*ZC3H3*	Zinc finger CCCH-type containing 3	1.09623
NM_001083113	*ZGPAT*	Zinc finger, CCCH-type with G patch domain	1.06153
NM_153608	*ZNF114*	Zinc finger protein 114	1.03268
NM_145911	*ZNF23*	Zinc finger protein 23	1.11284
NM_001136036	*ZNF692*	Zinc finger protein 692	1.1641
NM_030767	*AKNA*	AT-hook transcription factor	-1.34884
NM_001030287	*ATF3*	Activating transcription factor 3	-1.11158
NM_001193646	*ATF5*	Activating transcription factor 5	-2.18183
NM_001252296	*CEBPG*	CCAAT/enhancer binding protein (C/EBP), gamma	-1.18295
NM_001139514	*DACH2*	Dachshund family transcription factor 2	-1.86629
NM_001195053	*DDIT3*	DNA-damage-inducible transcript 3	-1.72535
NM_001278208	*DMC1*	DNA meiotic recombinase 1	-1.95125
NM_001160045	*GLI1*	GLI family zinc finger 1	-1.37881
NM_001546	*ID4*	Inhibitor of DNA binding 4, dominant negative helix-loop-helix protein	-1.65549
NM_001130172	*MYB*	V-Myb avian myeloblastosis viral oncogene homolog	-1.19446
NM_005761	*PLXNC1*	Plexin C1	-1.43667
NM_018699	*PRDM5*	PR domain containing 5	-1.29921
NM_001282116	*RFX3*	Regulatory factor X, 3 (influences HLA class II expression)	-1.13987
NM_001015881	*TSC22D3*	TSC22 domain family, member 3	-1.33723
NM_024620	*ZNF329*	Zinc finger protein 329	-1.33268
NM_020813	*ZNF471*	Zinc finger protein 471	-1.30429
NM_001007169	*ZNF483*	Zinc finger protein 483	-1.25458
NM_001204835	*ZNF568*	Zinc finger protein 568	-1.24492
NM_001172677	*ZNF607*	Zinc finger protein 607	-1.19447
NM_001077349	*ZNF682*	Zinc finger protein 682	-1.25533
NM_001037232	*ZNF829*	Zinc finger protein 829	-1.12351
NCCIT vs. EB+EtOH			
NM_002460	*IRF4*	Interferon regulatory factor 4	1.13354
NM_002228	*JUN*	JUN oncogene	1.85364
NM_001170703	*MBNL3*	Muscle blind-like 3 (Drosophila)	1.42077
NM_001024937	*MINK1*	Misshapen-like kinase 1 (zebrafish)	1.10139
NM_004529	*MLLT3*	Myeloid/lymphoid or mixed-lineage leukemia (trithorax homolog, Drosophila); translocated to, 3	1.0486
NM_020062	*SLC2A4RG*	SLC2A4 regulator	1.14312
NM_004176	*SREBF1*	Sterol regulatory element binding transcription factor 1	1.00315
NM_003220	*TFAP2A*	Transcription factor AP-2 alpha (activating enhancer binding protein 2 alpha)	1.34298
NM_007068	*DMC1*	DMC1 dosage suppressor of mck1 homolog, meiosis-specific homologous recombination (yeast)	-1.02572
NM_001033082	*MYCL1*	V-Myc myelocytomatosis viral oncogene homolog 1, lung carcinoma derived (avian)	-1.18131
NM_022367	*SEMA4A*	SEMA domain, immunoglobulin domain (Ig), transmembrane domain (TM) and short cytoplasmic domain, (semaphorin) 4A	-1.11894
NM_001145671	*SORBS2*	Sorbin and SH3 domain containing 2	-2.23762
NM_001010879	*ZIK1*	Zinc finger protein interacting with K protein 1 homolog (mouse)	-1.02557
NM_182609	*ZNF677*	Zinc finger protein 677	-1.61246
NM_032805	*ZSCAN10*	Zinc finger and SCAN domain containing 10	-1.04282
EB vs. EB+EtOH			
NM_001248002	*ARNTL2*	Aryl hydrocarbon receptor nuclear translocator-like 2	1.04288
NM_001030287	*ATF3*	Activating transcription factor 3	1.17257
NM_001193646	*ATF5*	Activating transcription factor 5	1.92788
NM_001139514	*DACH2*	Dachshund family transcription factor 2	1.37552
NM_001195053	*DDIT3*	DNA-damage-inducible transcript 3	1.08653
NM_001278208	*DMC1*	DNA meiotic recombinase 1	1.72917
NM_001160045	*GLI1*	GLI family zinc finger 1	1.03809
NM_001546	*ID4*	Inhibitor of DNA binding 4, dominant negative helix-loop-helix protein	1.34443
NM_001170701	*MBNL3*	Muscleblind-like splicing regulator 3	1.65711
NM_005761	*PLXNC1*	Plexin C1	1.11354
NM_018699	*PRDM5*	PR domain containing 5	1.00847
NM_145715	*TIGD2*	Tigger transposable element derived 2	1.14213
NM_001267597	*ZNF248*	Zinc finger protein 248	1.11911
NM_024697	*ZNF385D*	Zinc finger protein 385D	1.01064
NM_001077349	*ZNF682*	Zinc finger protein 682	1.1168
NM_001171166	*CAMTA2*	Calmodulin binding transcription activator 2	-1.55767
NM_021008	*DEAF1*	DEAF1 transcription factor	-1.15032
NM_001114123	*ELK1*	ELK1, member of ETS oncogene family	-1.00771
NM_005524	*HES1*	Hes family bHLH transcription factor 1	-1.02254
NM_003865	*HESX1*	HESX homeobox 1	-1.56485
NM_001267039	*LARP7*	La ribonucleoprotein domain family, member 7	-1.06637
NM_001130101	*NR1H3*	Nuclear receptor subfamily 1, group H, member 3	-1.19987
NM_001270523	*OTX2*	Orthodenticle homeobox 2	-1.0476
NM_019896	*POLE4*	Polymerase (DNA-directed), epsilon 4, accessory subunit	-1.03574
NM_021128	*POLR2L*	Polymerase (RNA) II (DNA directed) polypeptide L, 7.6kDa	-1.26959
NM_001077497	*PRR3*	Proline rich 3	-1.15658
NM_001015051	*RUNX2*	Runt-related transcription factor 2	-6.79717
NM_001145670	*SORBS2*	Sorbin and SH3 domain containing 2	-1.72074
NM_001178078	*STAT6*	Signal transducer and activator of transcription 6, interleukin-4 induced	-1.32528
NM_030935	*TSC22D4*	TSC22 domain family, member 4	-1.34099
NM_001276373	*USF1*	Upstream transcription factor 1	-1.06427
NM_032792	*ZBTB45*	Zinc finger and BTB domain containing 45	-1.44569
NM_015117	*ZC3H3*	Zinc finger CCCH-type containing 3	-1.13058
NM_001145347	*ZNF576*	Zinc finger protein 576	-1.17224
NM_001282415	*ZSCAN10*	Zinc finger and SCAN domain containing 10	-1.68229

**Fig 5:**
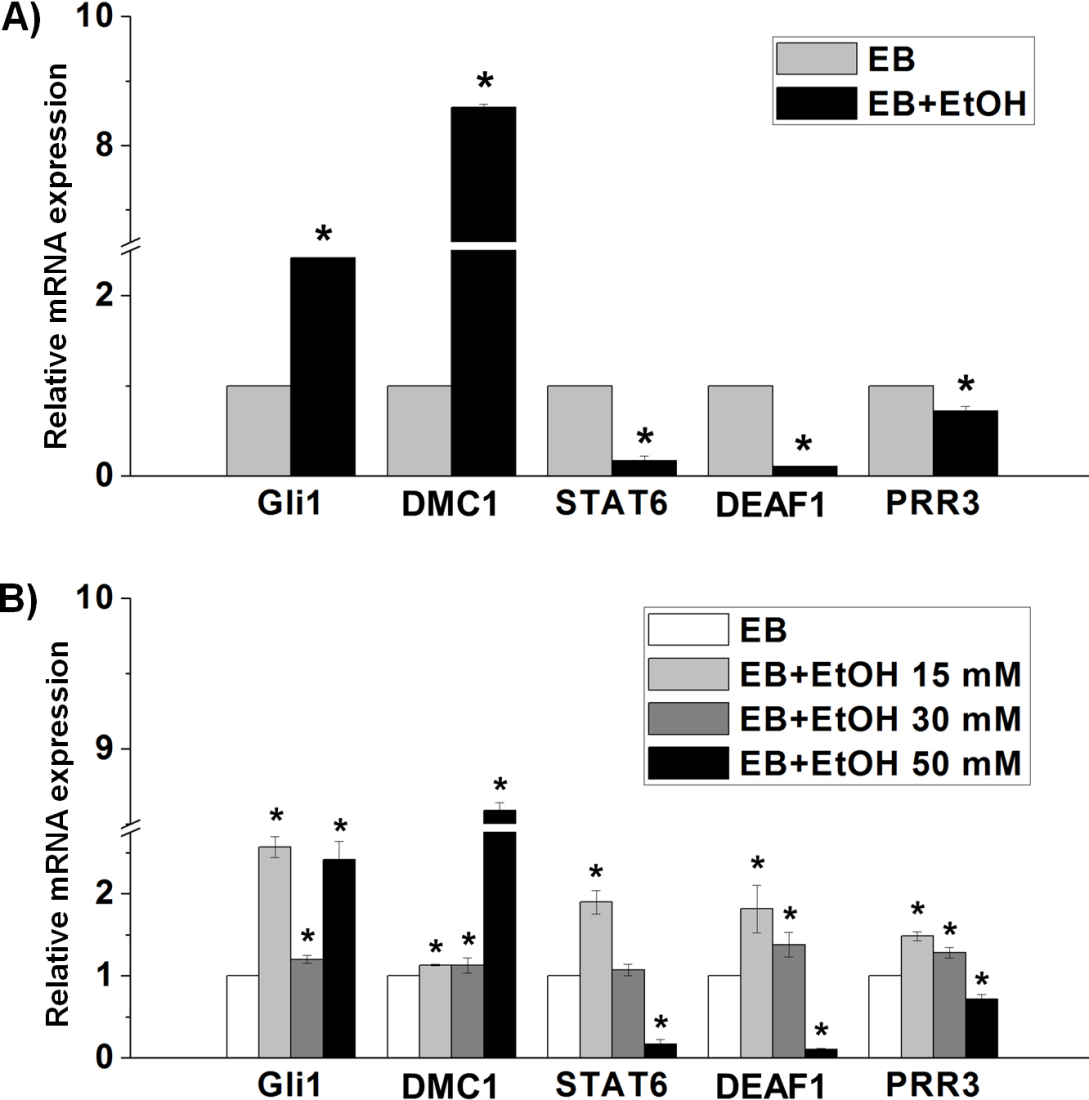
Confirmation of differential gene expression via qRT-PCR analysis. A) Validation of the relative mRNA expression of the TF-encoding genes that are randomly selected from the listed 35 genes found in EB vs. EB+EtOH dataset. B) Validation of the relative mRNA expression of the TF-encoding genes in different concentrations of ethanol. The expression value was normalized to the GAPDH expression level. Values are represented as average mRNA expression ± SEM bars, n = 3 replicates. Asterisks indicate statistically significant changes based on adjusted *p*-values < 0.05.

We also performed enrichment analysis of known TF motifs using known TF motifs in the JASPER database to identify motifs enriched in these differentially expressed TF-encoding genes. We found that there were a total of 80 TFs in NCCIT vs. EB, a total of 28 TFs in NCCIT vs. EB+EtOH and a total of 13 TFs in EB vs. EB+EtOH, whose binding sites were significantly over represented in the promoter region of differentially expressed TF-encoding genes (p <0.05). Top 5 enriched motifs are presented in Fig 4G, 4H and 4I. Our aim was to profile ethanol-responsive TFs that will help other researchers to find more molecular mechanism conducted by ethanol. So, we emphasized the TFs found in EB vs. EB+EtOH dataset and chose several genes randomly to validate their expressions. Expression graph showed the consistent results with RNA-seq analysis (Fig 5A). To observe the expression of selected TF-encoding genes in response with ethanol we also studied different concentrations of ethanol (15 mM, 30 mM and 50 mM) which mimic low, medium and high exposure units. qRT-PCR analysis showed that low and high doses were more effectual to developing EBs (Fig 5B).

## Discussion

Alcohol and developmental disorder is a well-established phenomenon but the manner in which alcohol exerts its toxic effect is still not well understood. Here, we have tried to explore genomic alterations mediated by ethanol during early development to profile ethanol-targeted gene expressions. In this study, we used NCCIT cells and applied RNA sequencing to profile ethanol-targeted genes and tried to categorize the genetic alterations according to molecular and cellular functions. We have listed a group of TFs that were regulated by ethanol during early development and, to our knowledge, we are the first group to profile a set of ethanol-responsive TFs. There is a similar study previously published [56]by our group where we tried to find proteomic changes by ethanol using MALTI-TOF MS. In the former study we treated NCCIT cells by ethanol from the very beginning without waiting for EB formation. In simple word, the formation of EB was under ethanol exposure itself. But in the recent study we did form EB at first and then exposed to ethanol. The later one is much closer to mimic embryonic state exposed by ethanol.

From the RNA sequencing data, we obtained a large number of genes that were differentially expressed. The number of altered genes highlighted a very simple analysis that ethanol is a potent regulator of gene expression, directly or indirectly. We can assume that the harmful effect of ethanol can be exerted through the genomic alterations due to its exposure at any time point and any stage of cellular development. From the GO analysis (S2 Fig) and disease pathway analysis (data not shown) of all DEGs, we notice several categories involved, such as neurological system processes, behavior, regulation of transcription and gene expression, cell adhesion, negative regulation of cell communication, neurological diseases, developmental disorders, skeletal and muscular disorders, psychological disorders and so on. It is noticeable that there may be a relationship between this alteration of genes and FASD symptoms, but studies into the exact molecular mechanism need to be conducted. However, our experimental model and obtained results are strong enough to correlate with alcohol-related genomic alterations and warrant more in-depth analysis.

It was found that ethanol accelerated the development of neurons during early development by altering expression of some important regulators when we analyzed EB vs. EB+EtOH. This was an interesting finding and we validated this prediction providing qRT-PCR analysis results of all 11 DEGs. From the previously reported articles we have found that those 11 genes were involved in regulation of neuronal morphogenesis, sprouting and branching of neurites, neuritogenesis, synaptogenesis, axonogenesis, length and branching of dendrites [41,42,43,44,45,46,47,48,49,50,51,52,53,54]. Thus, it can be assumed that ethanol is a potent regulator during early development. Whether the activation of neuronal development has any correlation with FASD seeks further in depth analysis.

Furthermore, we have selected TFs that have altered expression by ethanol exposure for one day. The relationship between ethanol and TF expression is not yet well studied. So, our provided list of TFs would be a very helpful asset in the study of early embryonic development in aspect of with or without ethanol. The mode of regulation is still unknown, and further in-depth analysis is needed. For further research it would be better to emphasized TFs from EB vs. EB+EtOH dataset because this is a direct comparison. We observed that among the 35 ethanol-targeted TFs found in EB vs. EB+EtOH dataset, a total of 8 belong to the zinc figure family. Zinc finger proteins are a large family of TFs involved in diverse functions, including DNA recognition, RNA packaging, transcriptional activation, the regulation of apoptosis, protein folding and assembly, and lipid binding [57]. The target genes and associated functions of the listed zinc finger proteins are not yet well characterized, but their presence is very much essential for normal development [58,59]. Studies suggest that altered zinc finger protein expression is associated with different diseases [60,61]. Ethanol, a potent teratogenic agent might have the same consequences during development as we found. Additional experiments are required to confirm this hypothesis. Here listed TFs could be treated as a marker gene to evaluate abnormal development under ethanol exposure. In a general sense, alteration of those TFs can cause different types of molecular and cellular abnormalities. Whether FASD-related abnormalities are due to altered expression of these TFs needs further investigation. We have found 4 TF-encoding genes which were common in NCCIT vs. EB+EtOH and EB vs. EB+EtOH data sets. We hope that these 4 TFs would be very meaningful to the future researchers to find ethanol-mediated molecular pathways by which it affects developmental dynamics.

Here, we provided a profile of genes that are altered by ethanol exposure during early development and selected a group of ethanol-targeted TFs. We did not validate the protein expressions of these TFs, which is a limitation of this study. To determine the target genes of these TFs and establish the association with ethanol-mediated abnormalities requires more in-depth analysis. We hope that our preliminary profile will help researchers in future studies and solve ethanol-mediated mysteries during fetal development.

## Supporting Information

S1 Fig Formation of EBs from NCCIT cells when cultured in suspension for 24 h. A) Morphology of EBs in phase contrast microscope. B) Immunocytochemical analysis for OCT4.
(DOCX)

S2 Fig Gene ontology analysis of all DEGs. A), B) and C) represent doughnut chart of the functional categories (Biological Process) of up- and down-regulated genes in NCCIT vs. EB, NCCIT vs. EB+EtOH and EB vs. EB+EtOH dataset, respectively. The top 15 significant categories are shown (*p*-values < 0.05). Numbers in the charts represent the relative percentage of total DEGs.
(DOCX)

S1 Table Read count for each experimental group and replicate obtained from RNA sequencing. (DOCX)

S2 Table List of Primer sequences used for validation of RNA-seq results. (DOCX)

S3 Table Top 15 enriched canonical pathways of all differentially expressed genes in NCCIT vs. EB datasets. (DOCX)

S4 Table Top 15 enriched canonical pathways of all differentially expressed genes in the NCCIT vs. EB+EtOH datasets. (DOCX)
