# Supplementary material for: RNA Sequencing Reveals the Alteration of the Expression of Novel Genes in Ethanol-Treated Embryoid Bodies
Source: PLoS One. 2016 Mar 1;11(3):e0149976. doi: 10.1371/journal.pone.0149976 (PMC4773011; doi:10.1371/journal.pone.0149976)
Supplement: S1 Fig — A) Morphology of EBs in phase contrast microscope. B) Immunocytochemical analysis for OCT4. (DOCX) [file pone.0149976.s001.docx]

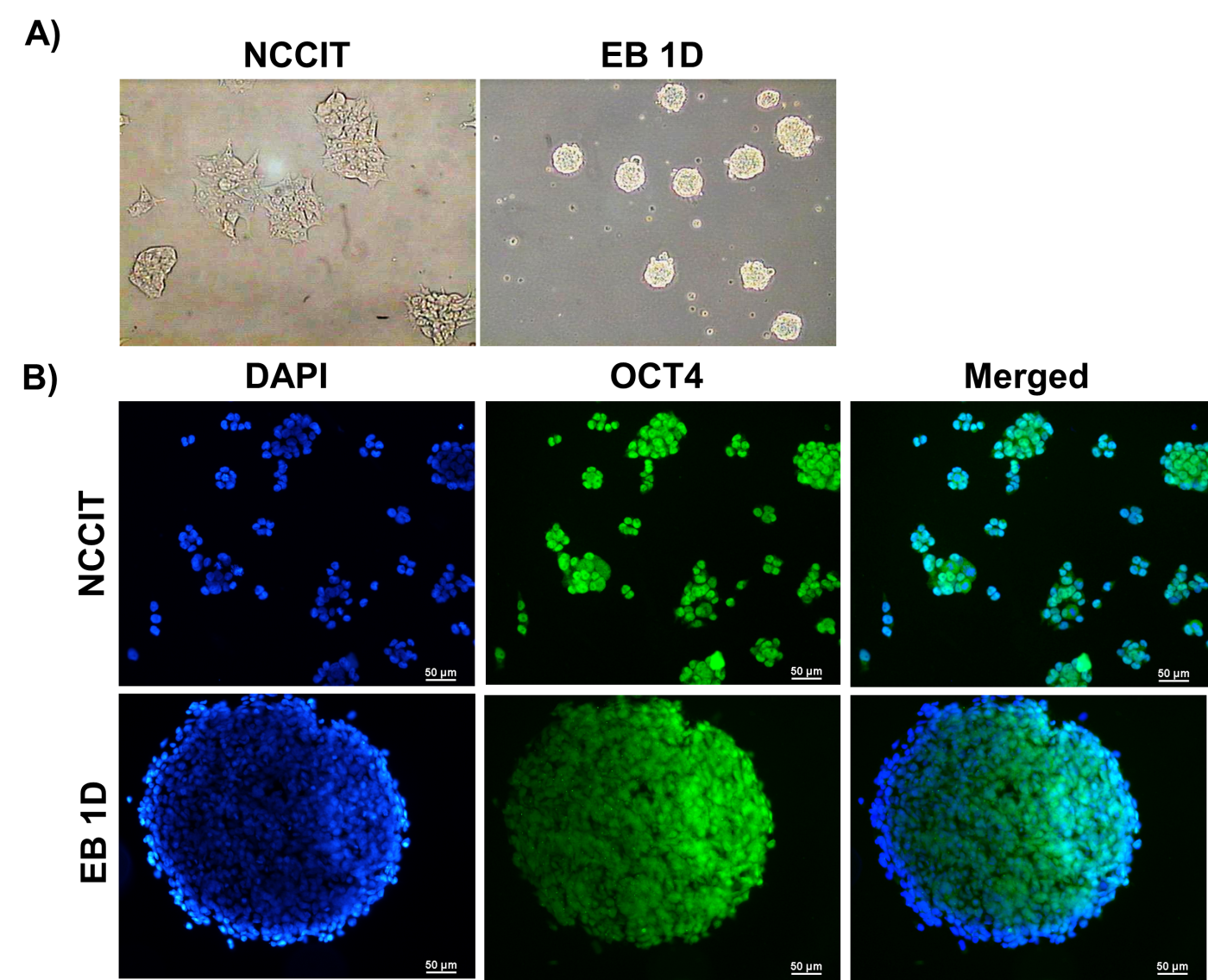


**S1 Fig.** **Formation of EBs from NCCIT cells when cultured in suspension for 24 h.** **A)** Morphology of EBs in phase contrast microscope. **B)** Immunocytochemical analysis for OCT4.
