# Supplementary material for: RNA Sequencing Reveals the Alteration of the Expression of Novel Genes in Ethanol-Treated Embryoid Bodies
Source: PLoS One. 2016 Mar 1;11(3):e0149976. doi: 10.1371/journal.pone.0149976 (PMC4773011; doi:10.1371/journal.pone.0149976)
Supplement: S2 Fig — A), B) and C) represent doughnut chart of the functional categories (Biological Process) of up- and down-regulated genes in NCCIT vs. EB, NCCIT vs. EB+EtOH and EB vs. EB+EtOH dataset, respectively. The top 15 significant categories are shown (p-values < 0.05). Numbers in the charts represent the relative percentage of total DEGs. (DOCX) [file pone.0149976.s002.docx]

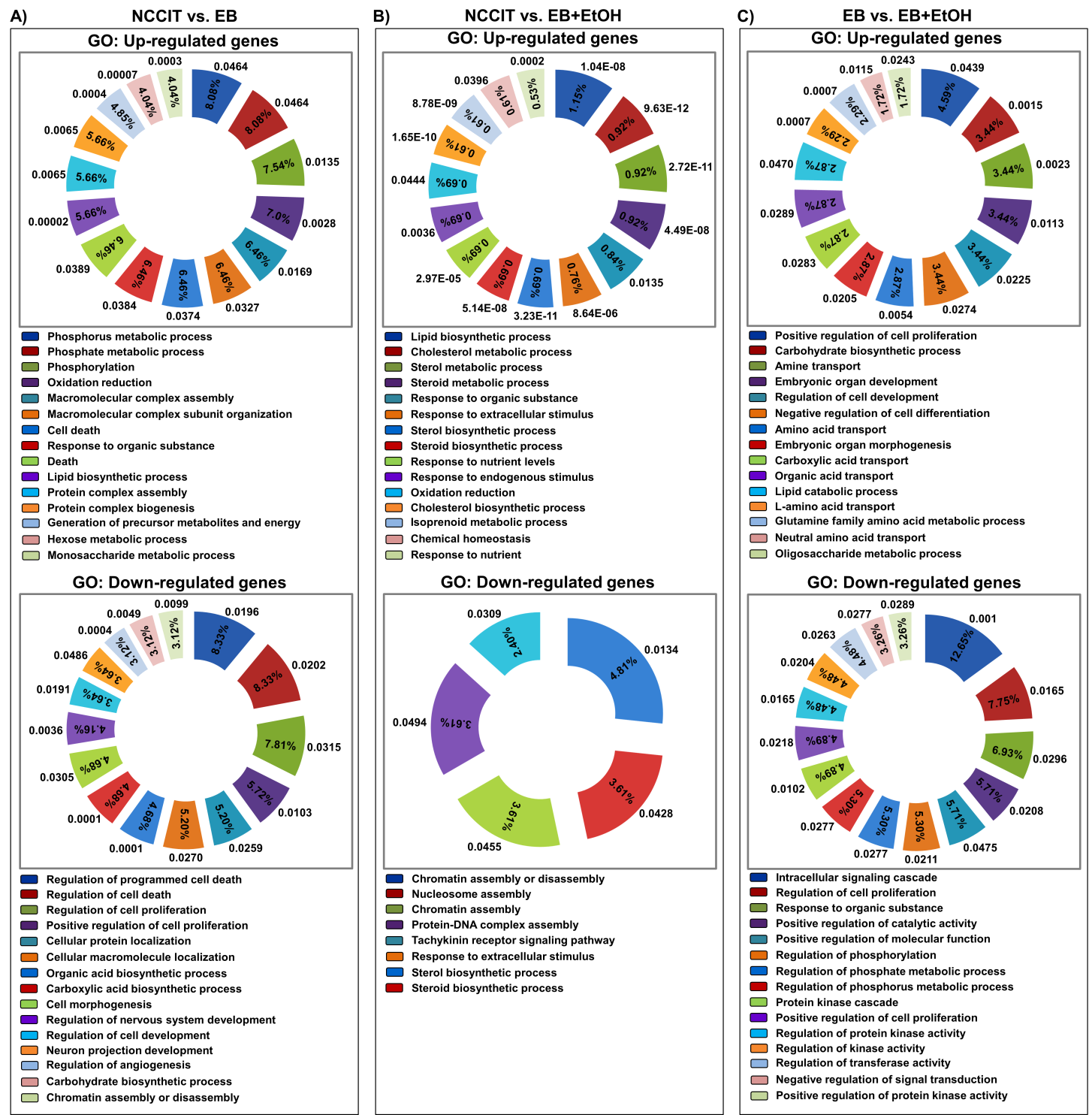


**S2 Fig. Gene ontology analysis of all DEGs.** A), B) and C) represent doughnut chart of the functional categories (Biological Process) of up- and down-regulated genes in NCCIT vs. EB, NCCIT vs. EB+EtOH and EB vs. EB+EtOH dataset, respectively. The top 15 significant categories are shown (p values < 0.05). Numbers in the charts represent the relative percentage of total DEGs.
