# Supplementary material for: RNA Sequencing Reveals the Alteration of the Expression of Novel Genes in Ethanol-Treated Embryoid Bodies
Source: PLoS One. 2016 Mar 1;11(3):e0149976. doi: 10.1371/journal.pone.0149976 (PMC4773011; doi:10.1371/journal.pone.0149976)
Supplement: S1 Table — (DOCX) [file pone.0149976.s003.docx]

**S1 Table.** **Read count for each experimental group and replicate obtained from RNA sequencing.**

| Replicates | Raw FASTQ files | |
| --- | --- | --- |
|  | Total reads | Overall alignment rate |
| NCCIT_1 | 17830200 | 79.53% |
| NCCIT_2 | 16260254 | 80.32% |
| EB_1 | 16420893 | 85.25% |
| EB_2 | 15942239 | 86.08% |
| EB+EtOH_1 | 19621735 | 79.01% |
| EB+EtOH_2 | 18819111 | 80.10% |
