# Supplementary material for: RNA Sequencing Reveals the Alteration of the Expression of Novel Genes in Ethanol-Treated Embryoid Bodies
Source: PLoS One. 2016 Mar 1;11(3):e0149976. doi: 10.1371/journal.pone.0149976 (PMC4773011; doi:10.1371/journal.pone.0149976)
Supplement: S2 Table — (DOCX) [file pone.0149976.s004.docx]

**S2 Table.** **List of Primer sequences used for validation of RNA-seq results.**

| Gene symbol | Sequences (5’-3’) | | Annealing Temperature (^o^C) |
| --- | --- | --- | --- |
|  | Forward | Reverse |  |
| *ATF3* | TTGCCATCCAGAACAAGCAC | TCTCCGACTCTTTCTGCAGG | 60 |
| *DDAH1* | CAAAAGGACAAATCAACGAGGTG | TGTGCAGATTCACTAGACCCAA | 60 |
| *PCYT1B* | CCATGAAAAACTGACCATTGCTC | GCATAAGGGCTCTTGCATGAC | 60 |
| *PRKCSH* | TCAGGTCAACGATGACTATTGC | CCCGGTTGGAGGGGATATACA | 60 |
| *DAB1* | TGCTGTGACCCAATTAGAACTTT | GCAACGTAACCTGAGGGTACA | 60 |
| *RHOB* | ATCCCCGAGAAGTGGGTCC | CGAGGTAGTCGTAGGCTTGGA | 60 |
| *GLI1* | TCA AGT TGA CCA AGA AGC GG | CTC ATG GTG CCA ATG GAG AG | 60 |
| *DMC1* | AGAAACATGGAATTAACGTGGCT | AAATGCAGTCAAGAATCCTGGTT | 60 |
| *STAT6* | AAGACCTGTCCATTCGCTCA | CATCTGGAGCTCTGGGGTAG | 60 |
| *DEAF1* | ACAGCCTGAACACCGAAAAAG | CACTGTCGTACACAGAAGGGT | 60 |
| *PRR3* | TAGAGGCCCAATTCGGAGAGG | GAGCCTTCGAGGGTTTCTCTG | 60 |
| *ELK1* | TGTTACCTCCACCATGCCAA | AGACTGGATGGTGAAGGTGG | 60 |
| *NFKBIA* | CTCCGAGACTTTCGAGGAAATAC | GCCATTGTAGTTGGTAGCCTTCA | 60 |
| *HES1* | ATT CCT CGT CCC CGG TGG CT | CAG CTT GGA ATG CCG CGA GCT | 60 |
| *THY1* | ATGAAGGTCCTCTACTTATCCGC | GCACTGTGACGTTCTGGGA | 60 |
| *LRRN1* | ATCTCACGACAGCTTCCCAA | CCTTGGACATTCTGGGCAAC | 60 |
| *GAPDH* | ATGGGGAAGGTGAAGGTCG | GGGGTCATTGATGGCAACAATA | 60 |
