# Supplementary material for: RNA Sequencing Reveals the Alteration of the Expression of Novel Genes in Ethanol-Treated Embryoid Bodies
Source: PLoS One. 2016 Mar 1;11(3):e0149976. doi: 10.1371/journal.pone.0149976 (PMC4773011; doi:10.1371/journal.pone.0149976)
Supplement: S3 Table — (DOCX) [file pone.0149976.s005.docx]

**S3 Table. Top 15 canonical pathways of all differentially expressed genes in NCCIT vs. EB dataset.**

| Ingenuity Canonical Pathways | -log  (p-value) | Molecules |
| --- | --- | --- |
| Glycolysis I | 7.97E00 | *PGK1, GPI, TPI1, PGAM1, PKM, ALDOA, GAPDH, PFKP, ALDOC* |
| Superpathway of Cholesterol Biosynthesis | 7.48E00 | *MVD, FDPS, FDFT1, EBP, DHCR7, MVK, MSMO1, LSS, HMGCS1* |
| Gluconeogenesis I | 5.48E00 | *PGK1, GPI, PGAM1, ALDOA, GAPDH, MDH2, ALDOC* |
| Cholesterol Biosynthesis I | 4.8E00 | *FDFT1, EBP, DHCR7, MSMO1, LSS* |
| Cholesterol Biosynthesis II (via 24,25-dihydrolanosterol) | 4.8E00 | *FDFT1, EBP, DHCR7, MSMO1, LSS* |
| Cholesterol Biosynthesis III (via Desmosterol) | 4.8E00 | *FDFT1, EBP, DHCR7, MSMO1, LSS* |
| Sertoli Cell-Sertoli Cell Junction Signaling | 4.52E00 | *MAP2K7, TUBB3, RRAS, TUBB4B, ACTB, TUBG1, ACTN3, TUBB2A, SYMPK, CLDN6, OCLN, JUN, TUBB4A, PVRL1, JUP, ACTG1* |
| Germ Cell-Sertoli Cell Junction Signaling | 3.9E00 | *PAK4, MAP2K7, TUBB3, CFL1, RRAS, TUBB4B, ACTB, TUBG1, ACTN3, TUBB2A, TUBB4A, JUP, PIK3R2, ACTG1* |
| Epithelial Adherens Junction Signaling | 3.74E00 | *TUBB3, MYL6, TUBB4B, RRAS, ACTB, TUBG1, TUBB2A, ACTN3, PVRL1, TUBB4A, JUP, TCF3, ACTG1* |
| Remodeling of Epithelial Adherens Junctions | 3.3E00 | *TUBB3, TUBB4B, ACTB, ACTN3, TUBB2A, TUBG1, TUBB4A, ACTG1* |
| Mechanisms of Viral Exit from Host Cells | 3.1E00 | *CHMP6, NEDD4, VPS28, ACTB, SH3GLB1, ACTG1* |
| Actin Cytoskeleton Signaling | 2.59E00 | *DIAPH2, PAK4, PFN1, MYL6, CFL1, FLNA, FGF2, RRAS, ACTB, ACTN3, PIK3R2, TMSB10, ACTG1, FGF19* |
| STAT3 Pathway | 2.43E00 | *PTPN6, RRAS, FGFR4, IGF1R, SOCS2, IGF2R, DDR1* |
| AMPK Signaling | 2.43E00 | *PFKFB3, PRKAB2, PCK2, SLC2A1, PFKFB4, CRTC2, FASN, ACTB, PPP2R5B, PRKAA2, PFKP, PIK3R2* |
| 14-3-3-mediated Signaling | 2.33E00 | *TUBB3, JUN, RRAS, TUBB4B, PLCG2, TUBB2A, TUBG1, TUBB4A, PIK3R2* |
