# Supplementary material for: RNA Sequencing Reveals the Alteration of the Expression of Novel Genes in Ethanol-Treated Embryoid Bodies
Source: PLoS One. 2016 Mar 1;11(3):e0149976. doi: 10.1371/journal.pone.0149976 (PMC4773011; doi:10.1371/journal.pone.0149976)
Supplement: S4 Table — (DOCX) [file pone.0149976.s006.docx]

**S4 Table. Top 15 canonical pathways of all differentially expressed genes in NCCIT vs. EB+EtOH dataset.**

| Ingenuity Canonical Pathways | -log  (p-value) | Molecules |
| --- | --- | --- |
| Super pathway of Cholesterol Biosynthesis | 1.35E01 | *MVD, FDPS, SQLE, FDFT1, DHCR7, IDI1, MVK, MSMO1, LSS, HMGCS1* |
| Cholesterol Biosynthesis I | 7.19E00 | *SQLE, FDFT1, DHCR7, MSMO1, LSS* |
| Cholesterol Biosynthesis II (via 24,25-dihydrolanosterol) | 7.19E00 | *SQLE, FDFT1, DHCR7, MSMO1, LSS* |
| Cholesterol Biosynthesis III (via Desmosterol) | 7.19E00 | *SQLE, FDFT1, DHCR7, MSMO1, LSS* |
| Super pathway of Geranylgeranyldiphosphate Biosynthesis I (via Mevalonate) | 6.52E00 | *MVD, FDPS, IDI1, MVK, HMGCS1* |
| HIF1α Signaling | 3.51E00 | *PIK3R3, JUN, SLC2A1, EGLN3, LDHA, MMP17* |
| LXR/RXR Activation | 3.12E00 | *SCD, FDFT1, LDLR, SREBF1, NGFR, SERPINF1* |
| Human Embryonic Stem Cell Pluripotency | 2.15E00 | *PIK3R3, WNT8A, PDGFA, TDGF1, ACVR1* |
| RAR Activation | 2.14E00 | *PIK3R3, DHRS3, LRAT, JUN, CYP26A1, CRABP1* |
| Role of Osteoblasts, Osteoclasts and Chondrocytes in Rheumatoid Arthritis | 1.85E00 | *PIK3R3, WNT8A, SPP1, JUN, NGFR, SFRP1* |
| Acute Phase Response Signaling | 1.75E00 | *PIK3R3, JUN, NGFR, SERPINF1, CRABP1* |
| Wnt/β-catenin Signaling | 1.75E00 | *WNT8A, CDH1, JUN, ACVR1, SFRP1* |
| Endothelin-1 Signaling | 1.72E00 | *PIK3R3, PLA2G6, JUN, GUCY2C, PNPLA3* |
| AMPK Signaling | 1.65E00 | *PIK3R3, PFKFB3, PCK2, SLC2A1, PFKFB4* |
| Role of Macrophages, Fibroblasts and Endothelial Cells in Rheumatoid Arthritis | 1.3E00 | *PIK3R3, WNT8A, JUN, PDGFA, NGFR, SFRP1* |
